# Bioinformatics analysis of diagnostic biomarkers for Alzheimer's disease in peripheral blood based on sex differences and support vector machine algorithm

**DOI:** 10.1186/s41065-022-00252-x

**Published:** 2022-10-04

**Authors:** Wencan Ji, Ke An, Canjun Wang, Shaohua Wang

**Affiliations:** 1 Nanjing Medical University, Nanjing, 211166 Jiangsu China; 2 Department of Endocrinology, Affiliated Zhongda Hospital of Southeast University, Nanjing, 210009 Jiangsu, China; 3 School of Medicine, Southeast University, Nanjing, 210009 Jiangsu China; 4 Department of Laboratory Medicine, Affiliated Zhongda Hospital of Southeast University, Nanjing, 210009 Jiangsu China

**Keywords:** Alzheimer's disease, Sex differences, Biomarker, SVM, Cluster analysis, Hub gene

## Abstract

**Background:**

The prevalence of Alzheimer's disease (AD) varies based on gender. Due to the lack of early stage biomarkers, most of them are diagnosed at the terminal stage. This study aimed to explore sex-specific signaling pathways and identify diagnostic biomarkers of AD.

**Methods:**

Microarray dataset for blood was obtained from the Gene Expression Omnibus (GEO) database of GSE63060 to conduct differentially expressed genes (DEGs) analysis by R software limma. Gene Ontology (GO) analysis, Kyoto Encyclopedia of Genes and Genomes (KEGG) pathway analysis and Gene set enrichment analysis (GSEA) were conducted. Immune checkpoint gene expression was compared between females and males. Using CytoHubba, we identified hub genes in a protein–protein interaction network (PPI). Then, we evaluated their distinct effectiveness using unsupervised hierarchical clustering. Support vector machine (SVM) and ten-fold cross-validation were used to further verify these biomarkers. Lastly, we confirmed our findings by using another independent dataset.

**Results:**

A total of 37 female-specific DEGs and 27 male-specific DEGs were identified from GSE63060 datasets. Analyses of enrichment showed that female-specific DEGs primarily focused on energy metabolism, while male-specific DEGs mostly involved in immune regulation. Three immune-checkpoint-relevant genes dysregulated in males. In females, however, these eight genes were not differentially expressed. SNRPG, RPS27A, COX7A2, ATP5PO, LSM3, COX7C, PFDN5, HINT1, PSMA6, RPS3A and RPL31 were regarded as hub genes for females, while SNRPG, RPL31, COX7C, RPS27A, RPL35A, RPS3A, RPS20 and PFDN5 were regarded as hub genes for males. Thirteen hub genes mentioned above was significantly lower in both AD and mild cognitive impairment (MCI). The diagnostic model of 15-marker panel (13 hub genes with sex and age) was developed. Both the training dataset and the independent validation dataset have area under the curve (AUC) with a high value (0.919, 95%CI 0.901–0.929 and 0.803, 95%CI 0.789–0.826). Based on GSEA for hub genes, they were associated with some aspects of AD pathogenesis.

**Conclusion:**

DEGs in males and females contribute differently to AD pathogenesis. Algorithms combining blood-based biomarkers may improve AD diagnostic accuracy, but large validation studies are needed.

**Supplementary Information:**

The online version contains supplementary material available at 10.1186/s41065-022-00252-x.

## Introduction

Alzheimer’s disease (AD) is the most prevalent neurodegenerative disease among the elderly, which is characterized by memory impairment, language, visuospatial skills and other cognitive domains decline [1]. It is reported that women have higher overall incidence than men [2–9]. Sexes differ in genetic drivers [10], clinical severity [11], and neuropathological manifestations [12, 13]. Focusing on the sex-specific AD genetic drivers could transform the way treatments are developed and administered and lead to more personalized interventions.

The primary pathologies of AD is associated with neurofibrillary tangles (NFTs), amyloid-β (Aβ) plaque deposition, inflammation, synaptic alterations, and neurovascular amyloidosis [14]. The sexual dimorphism in brain structure, genetic background, inflammation, gliosis, and immune module are considered as important implications for mechanistic investigation of AD [15, 16]. Men often have a greater brain volume than women, and so are less sensitive to pathological changes [17], such as atrophy, ATP synthase, the mitochondrial proteome, a redox protein, and cytochrome oxidase [18, 19]. Additionally, preclinical studies have demonstrated a significantly relationship between estrogen and soluble Aβ levels in the brains of wild-type mice after ovariectomy [20]. Sex differences also present in immune modulation. Women usually have stronger neuro-inflammation and neuro-immune response than men [21]. Some sex-specific genes are associated with AD pathogenesis such as amyloid and tau. For instance, females have a stronger correlation between APOE and tau than males [22]. In the GWAS study of CSF AD biomarkers, the female-specific roles played by SERPINB1 in amyloidosis, OSTN, and CLDN16 in tau pathology have been observed [23]. Another research has confirmed that a male-specific ubiquitin-specific peptidase 9 is a positive regulator of MAPT, a protein associated with AD [24]. As a multifactorial disease, biomarkers are crucial for accurate diagnosis, as well as aiding in the understanding of disease mechanisms. Fluid biomarkers may differ based on sex, but only a few studies have focused on this. Further research is needed.

**Fig. 1:**
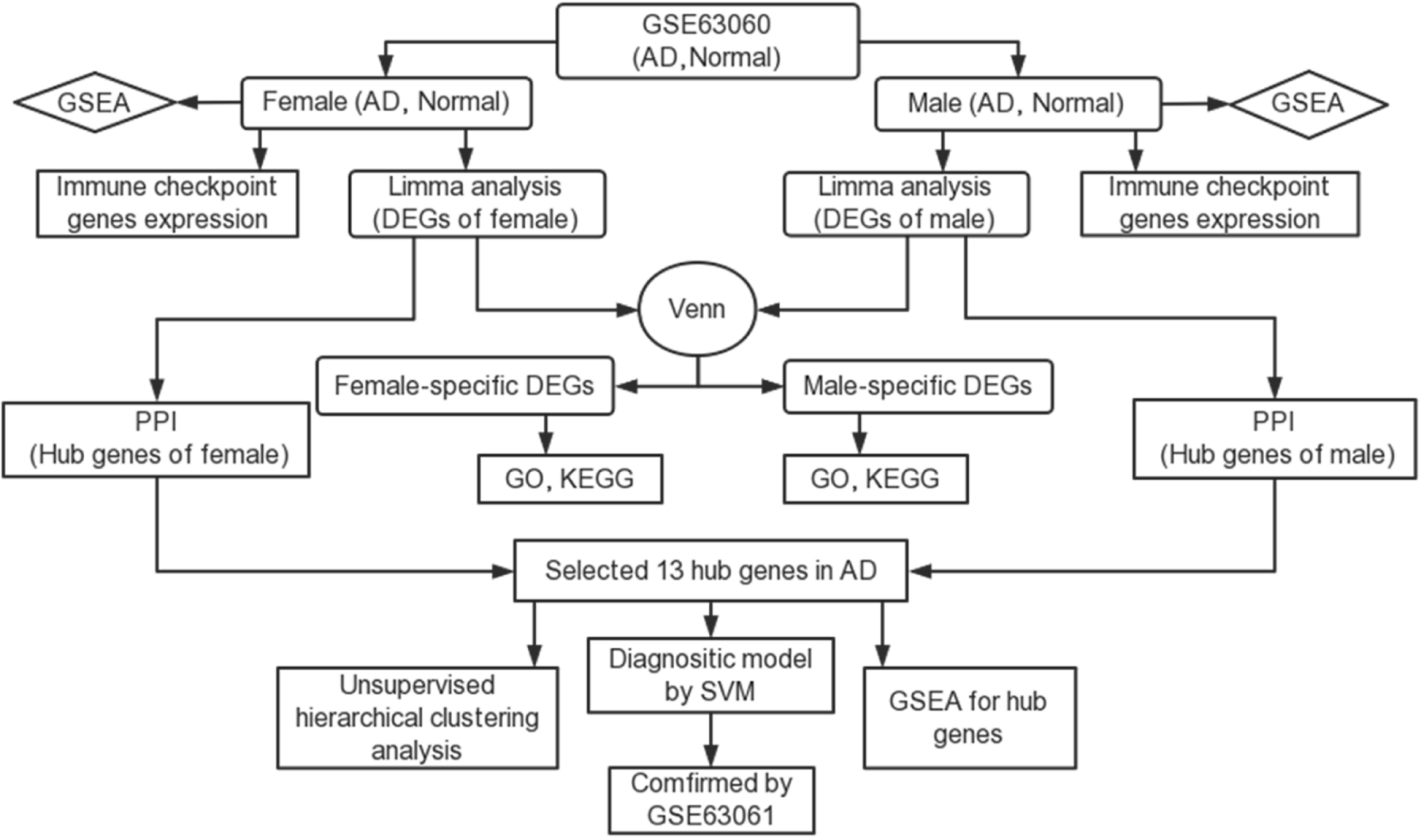
Work fow chart

Recently, with the development of the high-throughput sequencing, bioinformatics analysis is widely applied to unveil underlying mechanisms such as biomarkers identification or molecular classification of diseases [25, 26]. Our study is to investigate the critical sex-associated differentially expressed genes (DEGs) and identify novel diagnostic biomarkers. We first downloaded microarray datasets for peripheral blood in AD and control samples from the GEO database. Stratifying the total sample into two groups distinguished by sex. We identified DEGs among males and females separately. Next, series of enrichment analyses, protein–protein interaction (PPI) analysis, an unsupervised hierarchical clustering analysis and support vector machine (SVM) were performed. We identified hub genes in males and females separately. Combined with sex and age, a 15-gene-based diagnosis model was constructed. This study provides more molecular insights into the sex differences in AD, and identifies candidate biomarkers for diagnosis (Fig. 1).

## Materials and methods

### Microarray data acquisition and processing

We downloaded two expression profiling datasets from Gene Expression Omnibus (GEO, http://www.ncbi.nlm.nih.gov/geo). We filtered the datasets: 1) datasets with AD in Human Expression Profiling Using Arrays; 2) blood samples of AD; 3) each dataset contains at least five samples; 4) the research contains information about the technology and platform used. Finally, two microarray datasets GSE63060 [27] and GSE63061 [27] with were obtained (Table 1).

**Table 1:** Characteristics of the selected microarray datasets

Dataset	Platform	AD: MCI: Control	Age	female: male	Country
GSE63060	GPL6947	145: 78: 102	AD (58-88y)	198: 127	United Kingdom
			MCI (63-90y)		
			Control (52-87y)		
GSE63061	GPL10558	138: 109: 133	AD (59-95y)	231: 149	United Kingdom
			MCI (57-100y)		
			Control (63-91y)		

### Data normalization and identification of DEGs in males and females

Illumina expression chips from the bead series were used as the source of all data. LUMI package in R is used for data processing. Log_2_ processing is performed on raw data by lumiExpresso function. We used GSE63060 dataset to screen DEGs by comparing AD samples to Control samples in males and females separately. The download data format is MINIML. R package Limma (version: 3.40.2) [28] was used to analyze the mRNA differential expression. Adjusted *P*-values were calculated in order to correct for false positives. “Adjusted *P* < 0.05 and FC (Fold Change) > 1.3 or FC (Fold Change) <  − 1.3” were defined as the thresholds for the screening mRNAs for differential expression. The adjusted *P* value for multiple testing was calculated using the Benjamini–Hochberg correction. We constructed volcano plots based on fold-change values and adjusted P. The heat map is displayed by the R software package pheatmap. Using a Venn diagram, we identified and visualized the intersecting or sex-specific DEGs for males and females.

### Enrichment analysis and immune-checkpoint-relevant gene expression

Gene ontology (GO) and Kyoto Encyclopedia of Genes and Genomes (KEGG) pathway enrichment analyses were performed in R (version 3.6.3) with clusterProfiler (version 3.14.3) and calculated zscore by GO plot package [29] (version 1.0.2). These enrichment results were visualized using chord plots. Significantly enriched functions and pathways was screened of *p*-value < 0.05. Gene set enrichment analysis (GSEA) was used to analyze the distribution trend of the genes of a predefined set. GSEA was performed by package ggplot2 (Version 3.3.3) of R. The reference gene set is c2.cp.v7.2.symbols.gmt in the MsigDB database (https://www.gsea-msigdb.org/gsea/msigdb/collections.jsp). After 1,000 permutations, genes with an adjusted *p*-value < 0.05 are statistically significant [30].

Finally, we validated the immune-checkpoint-relevant gene expression in males and females separately. The statistical difference of two groups was compared through the Wilcox test.

### PPI network construction and hub gene analysis

To further explore the interactions among DEGs in males and females separately, PPI network analysis was performed using the online tool STRING (https://string-db.org/, version 11.5) [31] with a threshold of combination > 0.4. Cytoscape (version 3.8.2) (https://cytoscape.org/) was used to import the interaction information. Cytoscape's CytoHubba plugin was used to identify the hub genes. The top 20 hub genes were calculated using five algorithms including stress centrality, closeness centrality, radiality centrality, maximum neighborhood component (MNC), and degree. Finally, the hub genes were identified by intersecting the top 20 genes.

### Unsupervised hierarchical clustering analysis

To examine the effectiveness of hub genes in distinguishing AD and Control samples, an unsupervised hierarchical clustering analysis was performed. Consensus Cluster Plus R package (version v1.54.0) Clustering was used for clustering, six clusters are the maximum, and a total of 80% of the samples are drawn 100 times, clusterAlg = "hc", inner Linkage = 'ward.D2' [32]. Gene expression heatmaps with SD > 0.1 are maintained using the R software package pheatmap (version 1.0.12).

### Classification prediction of AD using SVM

An analysis of classification was conducted using Support Vector Machine (SVM) and Python (version 3.8). To reduce the effects of over fitting, our development and validation of a diagnostic model for AD includes a training step and a validation step. Most importantly, the model was evaluated using an independent data set only once. First, in each task of classification, radial basis function (RBF) were selected as kernel functions and cost (C) and gamma (γ) of the kernel function were found to be the best by a grid-search approach using ten-fold cross-validation and establishing receiver operation characteristic (ROC) curves. Finally, this machine learning model was evaluated by classifying AD in a completely independent validation set (GSE63061). Using the area under the receiver operating curve (AUC), the diagnostic performance was estimated. The diagnostic value of SVM classifier model was further verified by building an SVM with confusion matrix.

To recognize the biological process of the 13 hub genes that are possibly associated with AD in GSE63060 datasets, GSEA was performed again.

## Results

### Identification of DEGs

**Fig. 2:**
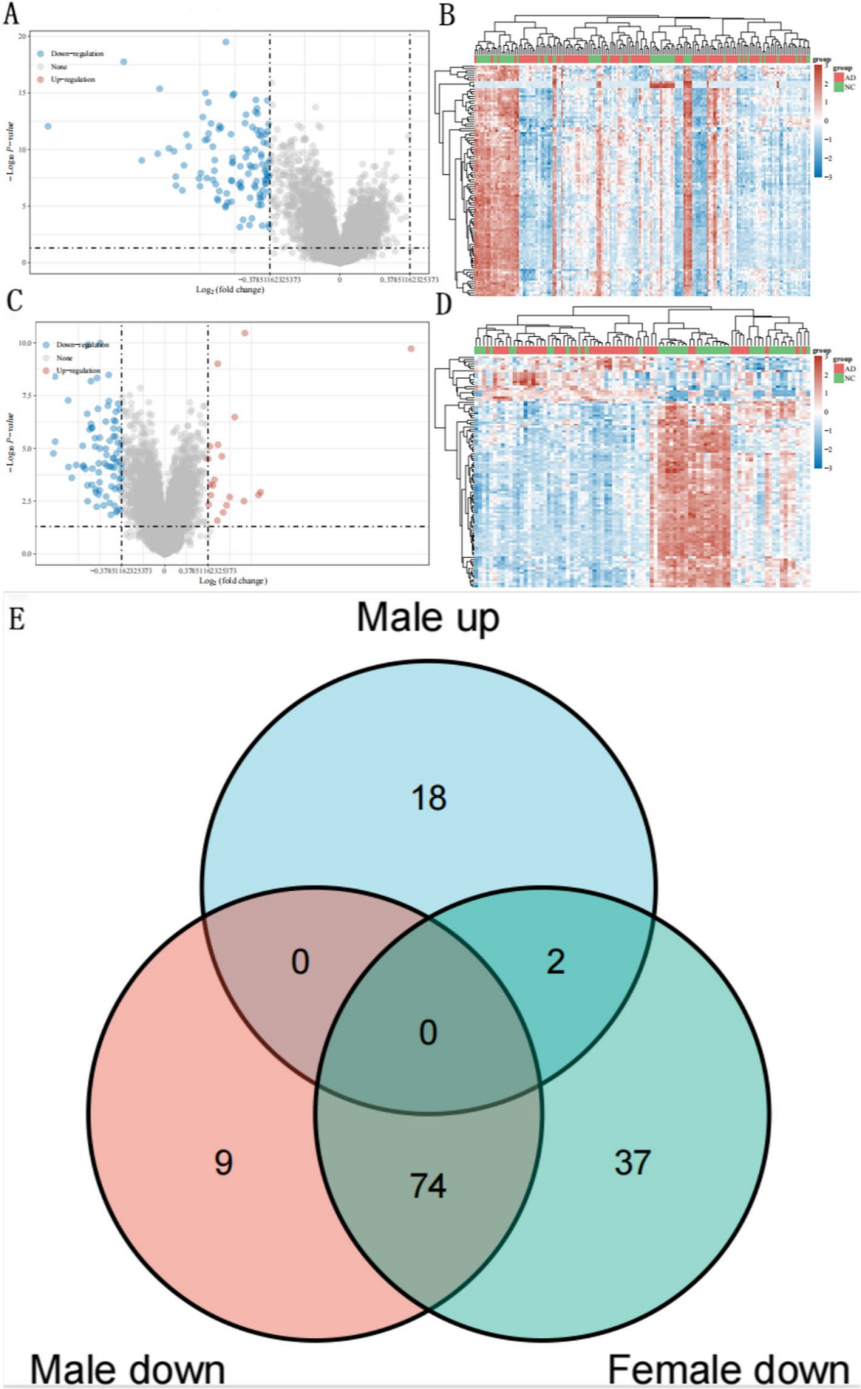
Identification of DEGs and venn diagram analysis in GSE63060. **A** Volcano plot of females. **B** Heatmap of females. **C** Volcano plot of males. **D** Heatmap of males. Volcano plot: red marks upregulated genes; grey marks non-significant genes; blue marks downregulated genes. Heatmap: red marks high expression; blue marks low expression. **E** Venn diagram of female-specific DEGs and male-specific DEGs. Light green: female-specific DEGs; orange and light blue: male-specific DEGs. AD: Alzheimer’s disease; NC: Normal control

The dataset GSE63060, which included 99 AD and 60 Control in females and 46 AD and 42 Control in males, were carried out to analyze the DEGs. We identified 113 downregulated DEGs in females (Fig. 2A, B and Supplementary Data 1), and 83 downregulated DEGs along with 20 upregulated DEGs were observed in males (Fig. 2C, D and Supplementary Data 2). Next, volcano plot and heatmap analyses were used to visualize these DEGs.

### Identification of sex-specific DEGs in males and females

We intersected DEGs lists of males and females from dataset GSE63060. Finally, 37 female-specific DEGs and 27 (18 + 9) male-specific DEGs were identified (Fig. 2E and Supplementary Data. 3).

### Enrichment analysis

**Fig. 3:**
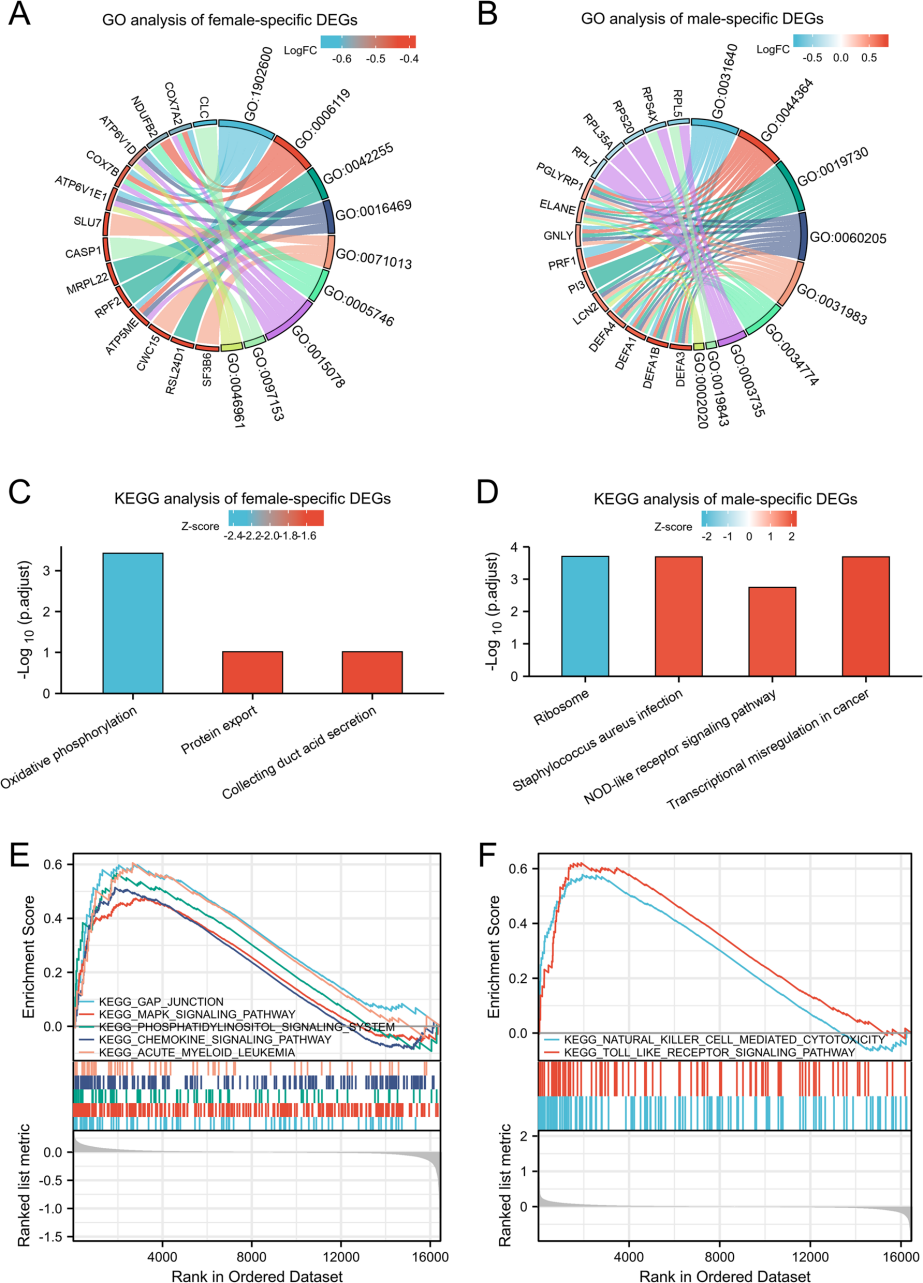
GO and KEGG pathway analyses of DEGs and GSEA plot. **A** The chord plot of DEGs in females. BP includes GO:1902600, GO:0006119, GO:0042255; CC includes GO:0016469, GO:0071013, GO:0005746; MF includes GO:0015078, GO:0097153, GO:0046961. **B** The chord plotof DEGs in males. BP includes GO:0031640, GO:0044364, GO:0019730; CC includes GO:0060205, GO:0031983, GO:0034774; MF includes GO:0003735, GO:0019843, GO:0002020. **C** KEGG in females. **D** KEGG in males. **E** GSEA in females. Female-specific pathway includes gap junction (NES = 1.800, p.adj = 0.036), acute myeloid leukemia (NES = 1.693, p.adj = 0.036), chemokine signaling pathway (NES = 1.677, p.adj = 0.040), phosphatidylinositol signaling system (NES = 1.649, p.adj = 0.036), and mapk signaling pathway (NES = 1.608, p.adj = 0.043). **F** GSEA in males. Male-specific pathway includes toll like receptor signaling pathway (NES = 1.875, p.adj = 0.038) and natural killer cell mediated cytotoxicity (NES = 1.819, p.adj = 0.038)

Analyses of GO and KEGG were used to investigate the functionalities of these sex-specific DEGs, respectively. GO analyses provide three different domain of biological processes (BP), molecular function (MF), and cellular component (CC). The top 3 GO analyses were selected and were drawn in a chord plot. Female-specific DEGs were mainly enriched in proton transmembrane transport, oxidative phosphorylation and ribosome assembly (BP); proton-transporting two-sector ATPase complex, catalytic step 2 spliceosome and mitochondrial respiratory chain(CC); proton transmembrane transporter activity, activity of cysteine-type endopeptidase in apoptosis, activity of proton-transporting ATPase and rotational mechanism (MF) (Fig. 3A and Supplementary Data 4). Male-specific DEGs tended to be enriched in killing of cells of other organism, disruption of cells of other organism and antimicrobial humoral response (BP); cytoplasmic vesicle lumen, vesicle lumen and secretory granule lumen (CC); structural constituent of ribosome, rRNA binding and protease binding (MF) (Fig. 3B and Supplementary Data 5). Next, we showed the KEGG pathways in the column chart. The KEGG pathway enrichment analysis in females showed that the DEGs were enriched in oxidative phosphorylation, protein export and collecting duct acid secretion (Fig. 3C and Supplementary Data 4). The KEGG pathway analysis in males revealed that the DEGs were significantly enriched in ribosome, transcriptional misregulation in cancer, staphylococcus aureus infection and NOD-like receptor signaling pathway (Fig. 3D and Supplementary Data 5).

In order to multi-perspective observation of the enrichment pathway, we will use GSEA to assess the genes' contribution to the phenotype. In both females and males, 21 and 16 pathways were enriched, respectively. Among them, there are 5 female-specific pathway and 2 male-specific pathway (Fig. 3E, F, Supplementary Data 6 and 7).

### Immune-checkpoint-relevant gene expression in males and females

**Fig. 4:**
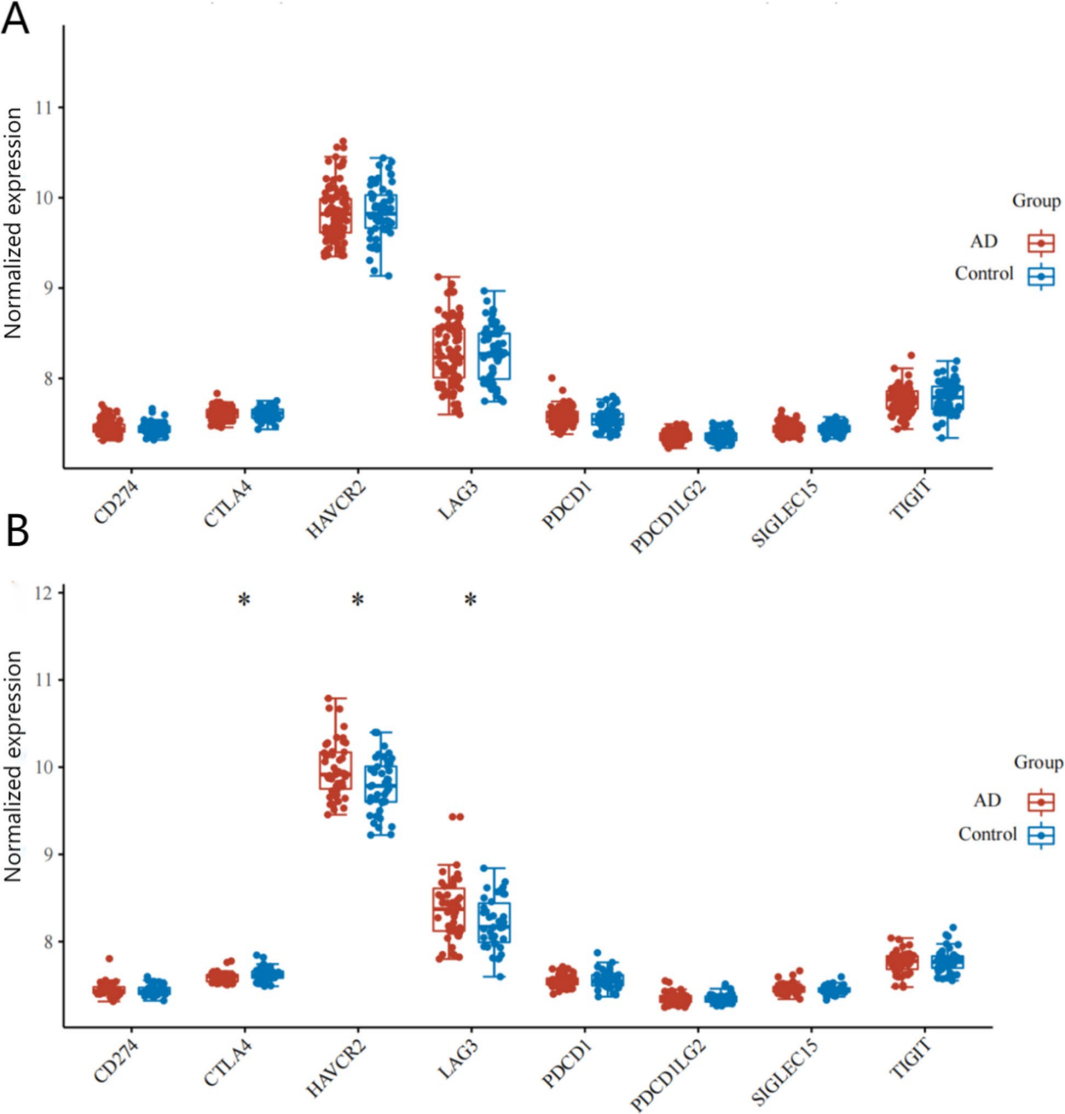
Analyse of genes related to immune checkpoints. The expression distribution of immune checkpoint in AD and Control. The abscissa represents different immune checkpoints; the ordinate represents Normalized expression level ( log_2_ transformation). *: *p* < 0.05. **A** Females; **B** Males

Immune checkpoint molecules are regulatory molecules that inhibit the immune system. Eight immune suppressive molecules were selected [33, 34]. Female and male immunocheckpoint-related genes were depicted through boxplots (Fig. 4). As compared with Control samples, HAVCR2 and LAG3 expression levels were significantly higher in male AD samples at 9.978 and 8.373 (*P* = 0.012 and 0.041; Wilcoxon rank sum test). CTLA4 was significantly lower in male AD samples at 7.592 (*P* = 0.032; Wilcoxon rank sum test). However, these eight genes were not differentially expressed in females (Supplementary Data 10).

### PPI network construction and hub gene selection

**Fig. 5:**
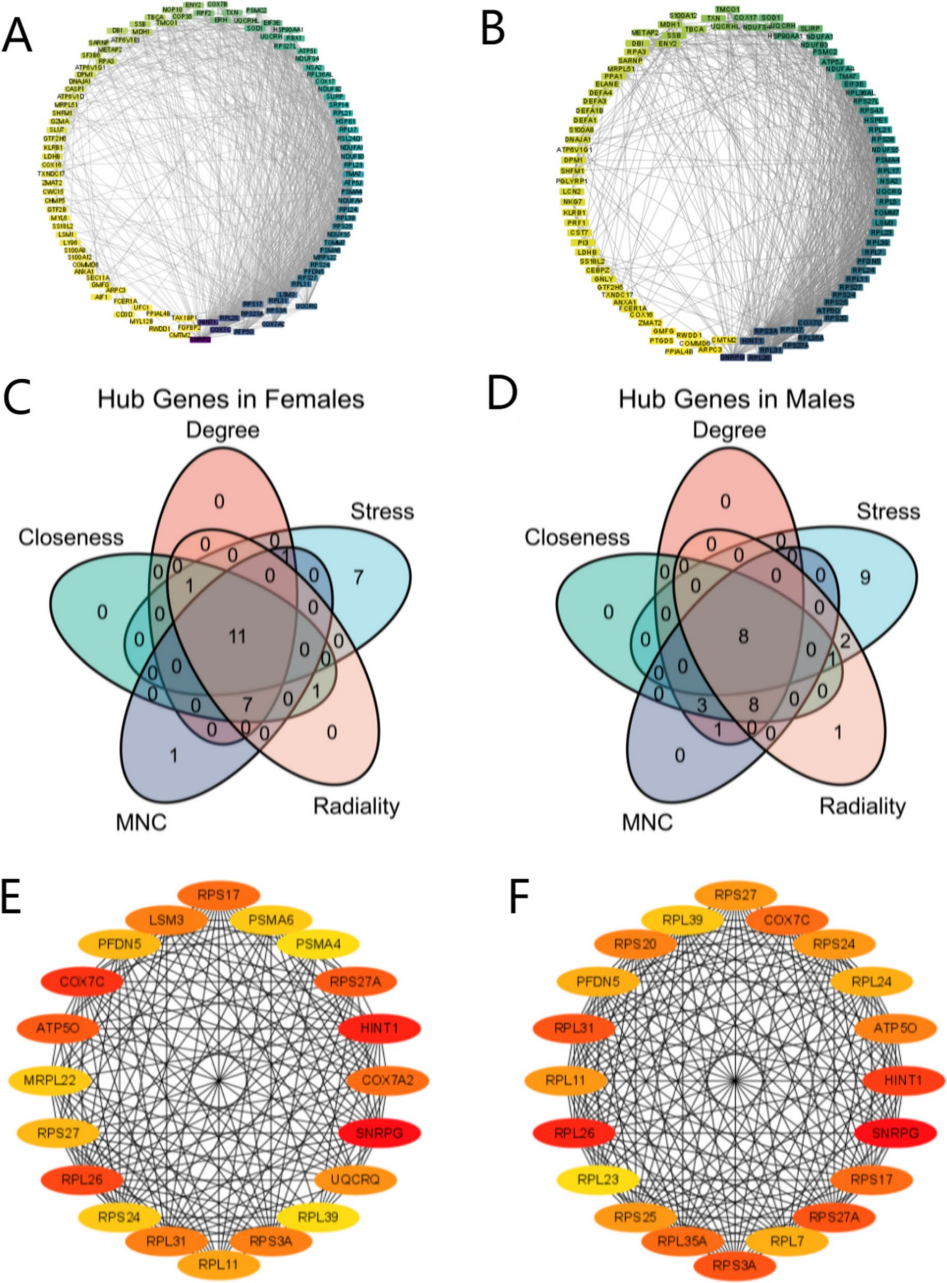
PPI network construction and hub gene selection. **A** PPI network of DEGs in females. **B** PPI network of DEGs in males. **C** Hub gene selection of females. **D** Hub gene selection of males. **E** The top 20 genes of degree in the female PPI network. **F** The top 20 genes of degree in the male PPI network. MNC: maximum neighborhood component

DEGs in females and males were inputted separately into STRING to obtain PPI records. The interaction network was visualized by Cytoscape 3.8.2 (Fig. 5A, B).

Next, the hub genes were identified with the cytoHubba plugin. According to the five algorithms of Degree, MNC, Radiality, Stress and Closeness, the top 20 hub genes were selected. Finally, eleven hub genes (SNRPG, RPS27A, COX7A2, ATP5PO, LSM3, COX7C, PFDN5, HINT1, PSMA6, RPS3A and RPL31) in females and eight hub genes (SNRPG, RPL31, COX7C, RPS27A, RPL35A, RPS3A, RPS20 and PFDN5) in males were identified (Fig. 5C, D, Supplementary Data 8 and 9).

To further identify the genetic evidence of sex differences, the top 20 genes by degree are shown separately for males and females (Fig. 5E, F). RPL39 is located on the X chromosome in males, while females lack the first 20 genes on the X chromosome.

### Hub gene expression in MCI and AD

**Fig. 6:**
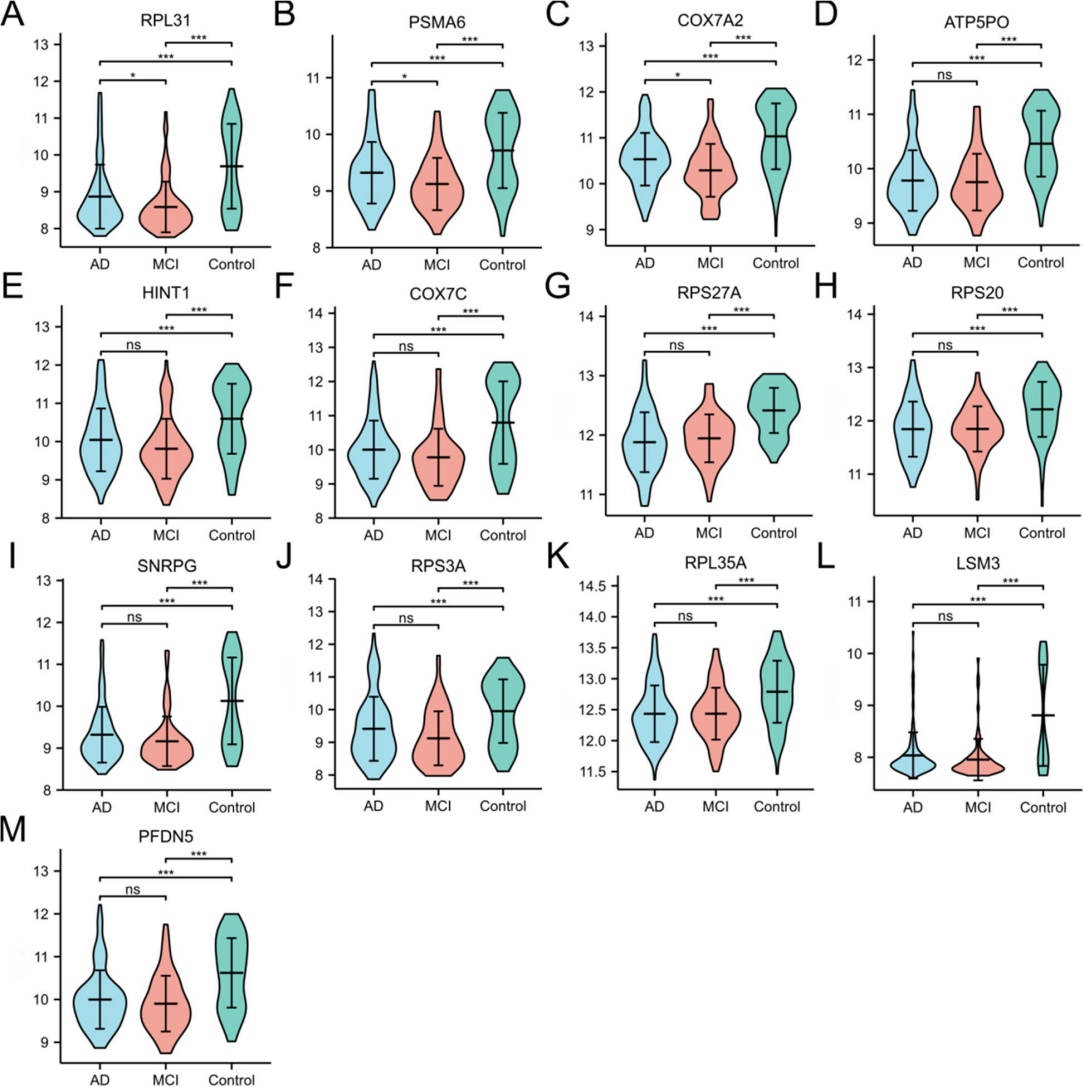
Expression levels of the 13 hub genes in MCI and AD by GSE63060. The abscissa represents AD, MCI and Control samples; the ordinate represents Normalized expression level. *: *p* < 0.05; **: *p* < 0.01; ***: *p* < 0.001; ns: no significant difference

Considering the remarkable expression changes of these 13 mRNAs (SNRPG, RPS27A, COX7A2, ATP5PO, LSM3, COX7C, PFDN5, HINT1, PSMA6, RPS3A, RPL31, RPL35A and RPS20) in male and female patients with AD separately, they may play a role in AD pathogenesis. Consequently, the 13 promising mRNAs were selected for further study. Using GSE63060, we assessed whether the expression levels of the 13 hub genes differed. Compared with the Control samples, the expression levels of the 13 genes were significantly decreased among the MCI and AD samples (*p* < 0.001; Dunn’s test). Additionally, we found that the mRNA expression levels of RPL31, PSMA6 and COX7A2 in the AD samples were higher than those in MCI samples (*p* = 0.042, 0.035 and 0.020; Dunn’s test) (Fig. 6 and Supplementary Data 10).

**Fig. 7:**
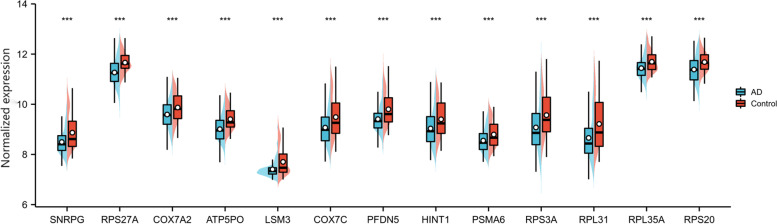
Expression levels of the 13 hub genes in GSE63061. The abscissa represents 13 hub genes; the ordinate represents Normalized expression level ***: *p* < 0.001

Our next step was to compare the 13 gene expression level between AD and Control samples by enrolling another independent dataset (GSE63061). As indicated in Fig. 7, AD samples show a significant reduction in the mRNA expression of all 13 hub genes (*p* < 0.001, Wilcoxon rank sum test) (Supplementary Data 10).

**Fig. 8:**
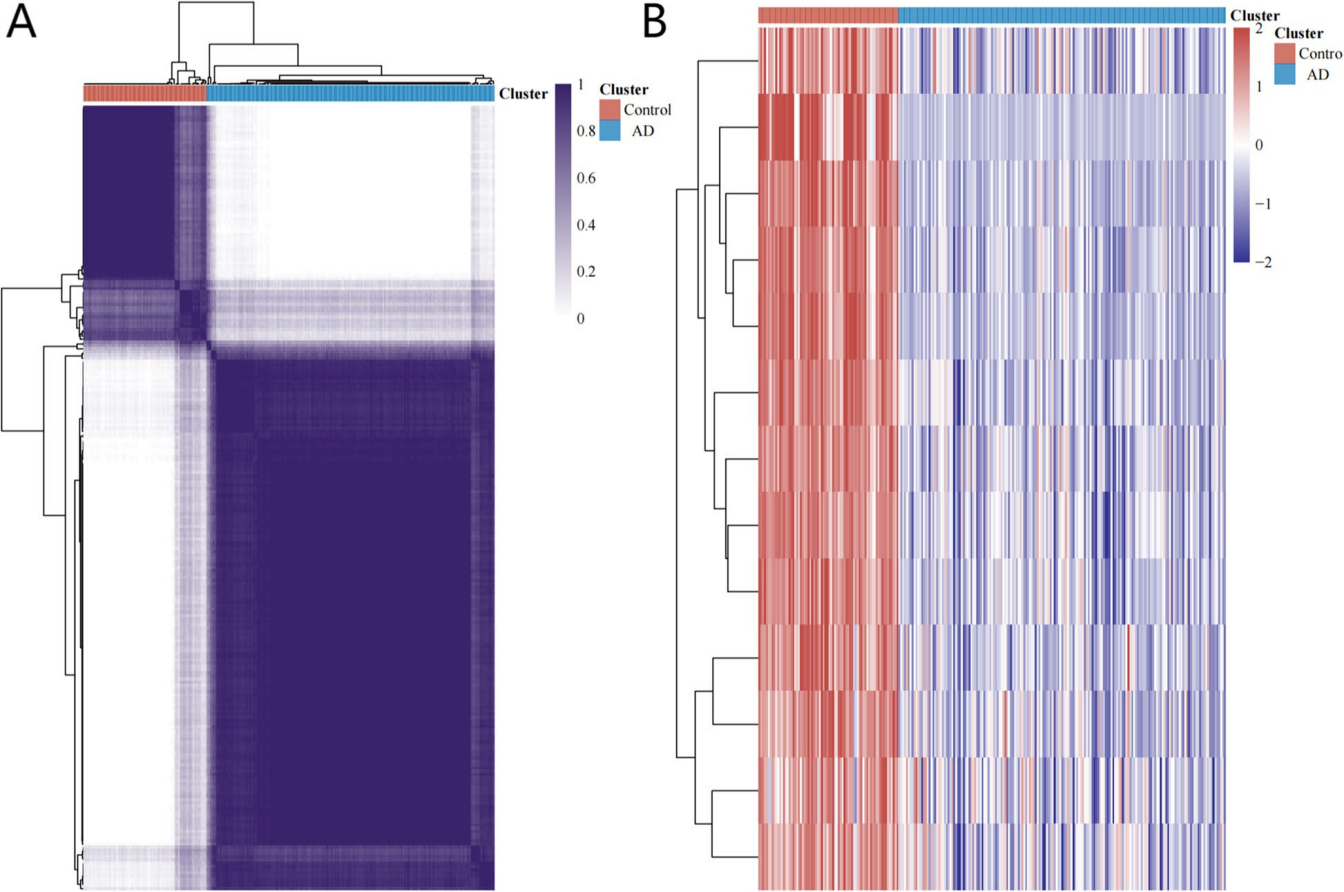
Unsupervised hierarchical clustering analysis. **A** Consistency of clustering results heatmap. Rows and columns represent samples; blue represents AD; red represents control. **B** The expression heatmap of 13 hub genes in AD and control; red represents high expression; blue represents low expression

Since no single mRNA has been shown to have a prominent diagnostic value to date, greater attention has been given to their synergistic effects. To demonstrate the classification ability of the 13 hub genes combination, an unsupervised hierarchical clustering analysis was performed. As shown in Fig. 8, these hub genes demonstrate an excellent capability for distinguishing samples with similar clinical manifestations.

### Identification of Potential Biomarkers of AD Using SVM

**Fig. 9:**
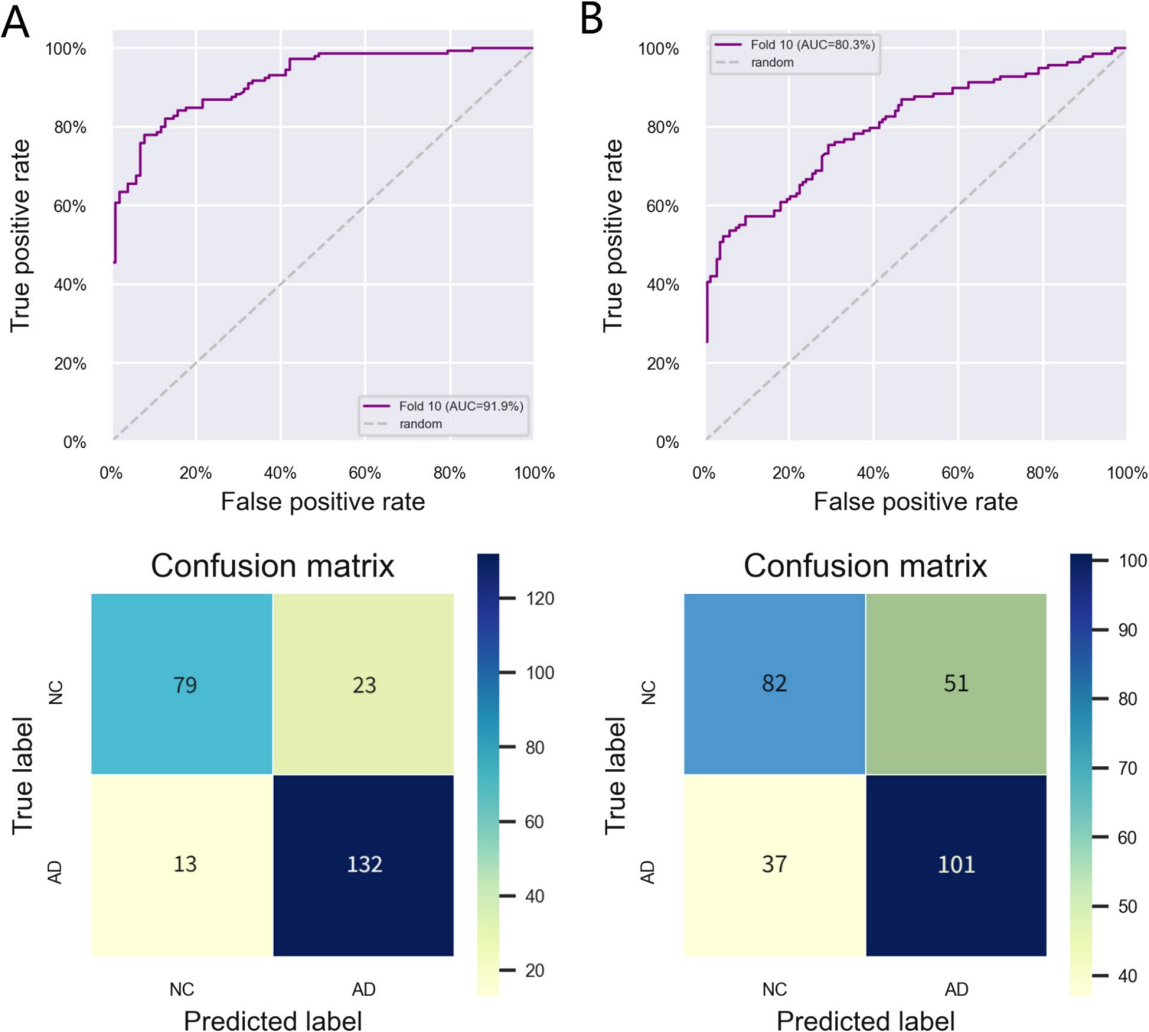
Support vector machine analysis of the 15-marker panel. **A** ROC curves of classification and confusion matrix in GSE63060. **B** ROC curves of classification and confusion matrix in GSE63061. AD: Alzheimer’s disease; NC: Normal control

According to the analyses above, we obtained different hub genes and performed enrichment analysis for females and males separately. Besides genes, clinical features such as age and sex may also play an important role in pathogenesis. Thus, we used the expression levels of these 13 genes combined with age and sex data as features to investigate their diagnostic value. SVM analysis with a ten-fold cross validation procedure indicated the AUC of the 15-marker panel was 0.919 (95%CI, 0.901–0.929). Furthermore, SVM with confusion matrix analysis also achieved an accuracy of 85.4%, with sensitivity of 91.0% and specificity of 77.5% (Fig. 9A).

A second microarray dataset GSE63061 was collected and analyzed using SVM in order to confirm the classification reliability of the above selected feature genes. As expected, the 15-marker panel can provide an AUC of 0.803 (95%CI, 0.789–0.826) and achieved an accuracy of 69.4%, with sensitivity of 73.2% and specificity of 61.7% (Fig. 9B).

**Fig. 10:**
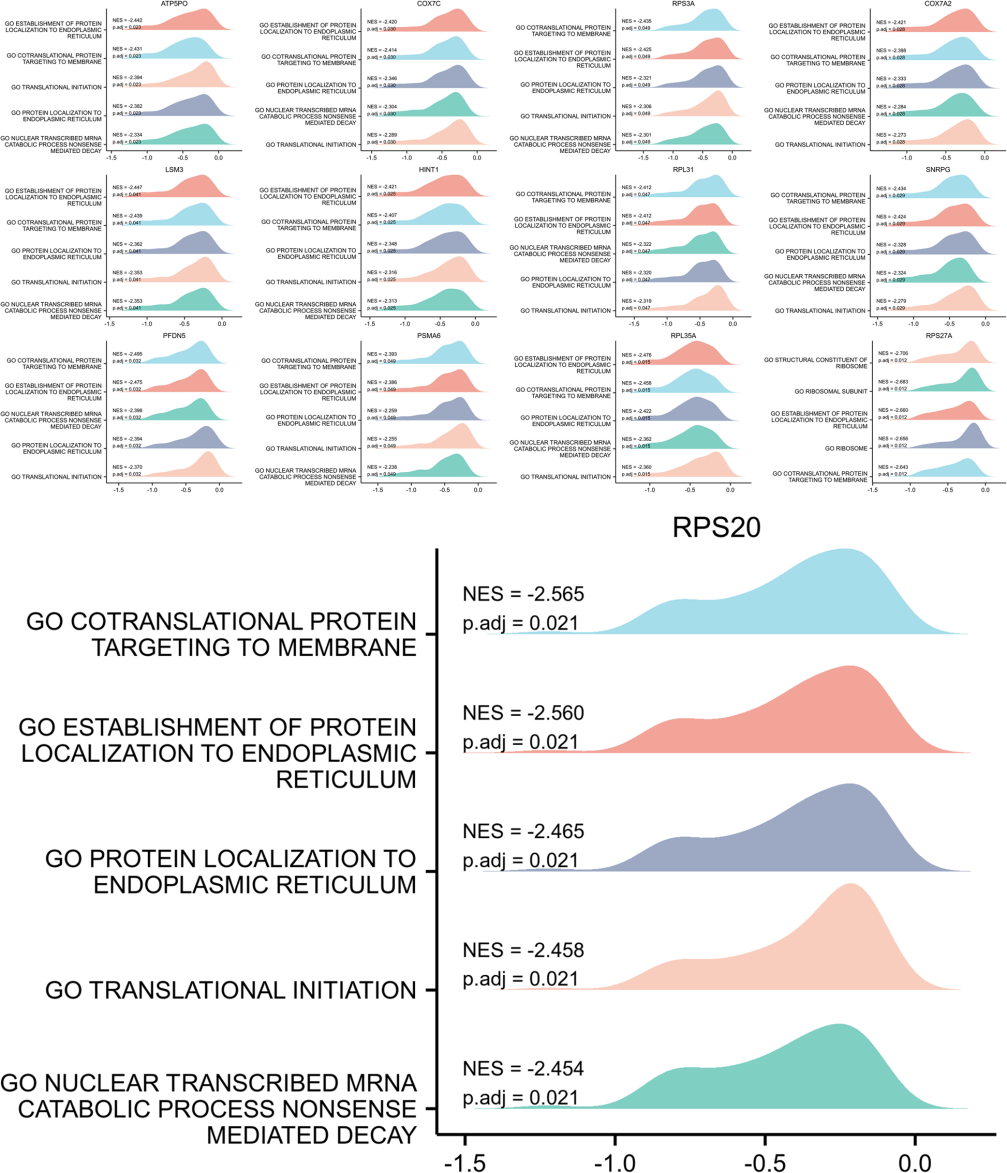
GESA for the 13 hub genes

Towards gaining deeper insights into the 13 hub genes, we identified potential BPs between AD and Control using GSEA. They are shown as a mountain map (Fig. 10). Most of these genes have been implicated in cotranslational protein targeting to membrane, establishment of protein lacalization to endoplasmic reticulum, protein localization to endoplasmic reticulum and translational initiation.

## Discussion

AD is a common disorder in the elderly and females are at higher risk of developing AD than males. Previous researches were usually based on CSF or brain tissue that may limit the application. Many studies have demonstrated that the blood can be used to detect disease-related changes [35–37]. Our study recruited blood tissue microarray data, and we integrated the differentially expressed genes in females and males separately.

Base on sex-specific DEGs, the KEGG pathways with smallest *p*-value enriched in females include oxidative phosphorylation, protein export, and collecting duct acid secretion. KEGG pathways enriched in males include ribosome, transcriptional misregulation in cancer, staphylococcus aureus infection, and NOD-like receptor signaling pathway. We discovered that abnormality of pathway is often associated with energy metabolism in females, whereas in males, the abnormality is primarily related to immune regulation. Females are more susceptible to estrogen's effects on energy homeostasis. Research has shown that estrogen in females plays a significant role in AD sex differences in preclinical and clinical studies [38, 39]. Female-specific pathways have also been found in a study of sex differences, but there were no male-specific pathways, possibly because tissue and ethnicities differ [40]. During AD pathogenesis, ribosome dysfunction can lead to altered translation, specifically in astrocytes [41, 42]. During immune challenge, male astrocytes produced more inflammatory molecules than female derived from cerebral cortex [43]. These studies show that males generally appear to have higher neuroimmune tone in the brain. However, the association between sex and these pathways involved in AD has not been studied. Additionally, analyzing the BP of male-specific DEGs showed that immune function, such as killing of cells of other organism, disruption of cells of other organism, antimicrobial humoral response, were stronger in AD samples.

To gain comprehensive insights, GSEA was conducted to identify potential pathways among males and females. As expected, male-specific pathway including toll like receptor signaling pathway and natural killer cell mediated cytotoxicity favours an immunological abnormalities. Next, we further detected the immune checkpoint molecules. Three of the eight immune checkpoint genes dysregulated in males, however, there were no differential gene expression in females. An immune checkpoint is a molecule that inhibits the immune system. Through controlling immune responses, they are essential to maintain self-tolerance and prevent autoimmune reactions. The observed sex dimorphism in immune checkpoint inhibitor responses is likely due to inherent differences between males and females [44–46]. Recent studies confirm that the immune checkpoint contributes to AD pathogenesis. It is well documented that mini-mental state examination (MMSE), tau proteins and Amyloid-β in AD were demonstrated to correlate with CTLA-4^+^T cells [47]. There could be sex-based differences in AD-intrinsic features that have been shaped by the immune system during AD development and immunoediting.

In the PPI network, SNRPG, RPS27A, COX7A2, ATP5PO, LSM3, COX7C, PFDN5, HINT1, PSMA6, RPS3A and RPL31 were identified as the female-specific hub genes, and SNRPG, RPL31, COX7C, RPS27A, RPL35A, RPS3A, RPS20 and PFDN5 were identified as the male-specific hub genes. SNRPG, RPS27A, COX7C, PFDN5, RPS3A and RPL31 were identified as common hubs. Nevertheless, other hub genes differ greatly between sexes. Moreover, the 13 genes were significantly dysregulated with AD progression.

A cluster of genes associated with the function and structural component of ribosomes, including RPS27A, RPL31, RPL35A, RPS3A, and RPS20, makes up most of the male-specific genes. Transcriptional changes in ribosome-related genes affect downstream translation in a broader way. Ribosome profiling was utilized in an in vitro experiment to investigate how the lack of oxygen and glucose immediately changes the transcription and translation of the genes in brain cells. The study concluded that oxidative stress has a greater effect on translation than transcription. It is alarming that testosterone can increase oxidative stress [48, 49] and oxidative stress-related conditions [50], and oxidative stress exacerbates testosterone's negative impact on cognition through its negative effects on androgens [51–54]. It can be inferred that sexually dimorphic differences may be a result of the expression of sex-specific ribosomal differential genes in AD. PSMA6 belongs to component of the 20S core proteasome complex that participates in the ATP-dependent degradation of ubiquitinated proteins. Ubiquitin proteasome system activity is integral to estradiol’s effects on memory, then this could lead to exciting new avenues of basic research into hormonal regulation of cognition, which could lead to important clinical implications for treating neurodegenerative disorders in which sex-based differences play a role [55]. LSM3 and SNRPG take role in the spliceosome's construction. As a component of the U4/U6-U5 tri-snRNP and the U1, U2, U4, and U5 snRNP complexes, they mostly performs pre-mRNA splicing-related tasks, including as RNA silencing and destruction. A snRNA(U1)accumulation has been observed in Alzheimer's disease [56]. In human brains, identifying cryptic splicing errors with neurofibrillary tangle burden implicates spliceosome disruption and transcriptome perturbation in AD Tau-mediated neurodegeneration [57]. These results show an independent function of U1 snRNA in regulating RNA splicing, suggesting that aberrant RNA processing may mediate neurodegeneration [58]. One study profiled the expression of snRNAs by applying small RNA sequencing to sncRNA isolated from anterior cingulate cortex of schizophrenia patients. Two snRNAs were found to be differentially expressed between female cases and controls [59]. However, no report has been published on the relationship between snRNA and sex differences in AD. HINT1 belongs to the histidine triad superfamily, which is widely expressed in different tissues. Ageing of the brain is a major risk factor for many neurodegenerative disorders including Alzheimer's. The downregulation of HINT1 has also been reported in diabetes and AD [60, 61]. Yu W et al. [62] found that potassium 2‐(1‐hydroxypentyl)‐benzoate can increase HINT1 expression levels, thus improving spatial learning and memory deficits in diabetic animals. Chen Q et al.'s study suggests that HINT1 may be associated with schizophrenia and the association is sex specific [63]. According to our study, HINT1 was found to be significantly differentially expressed in female AD. All these results suggest that HINT1 may play a role in neuronal function, but its exact physiological and cellular functions in AD remain unknown. PFDN5 belongs to prefoldin family. It is highly expressed in neurons and other neural cells, which can protect cells from apoptosis by decreases the toxicity of misfolded proteins. Interestingly, whole blood mRNA expression data from Alzheimer's patients revealed downregulation of PFDN5, which could be related to higher levels of toxicity of Aβ [64]. The results of our research analysis support this conclusion. The present study identified ATP5PO and COX7A2 as female-specific hub genes, which were significantly associated with ATP synthesis, heat production and oxidative phosphorylation. In female brains, estrogen control of glucose metabolism is dismantled during midlife, resulting in a shift in fuel systems and the emergence of dynamic neuroimmune phenotypes. As fuel use shifts, white matter is at risk of catabolism [65]. PSMA6 belongs to component of the 20S core proteasome complex that participates in the ATP-dependent degradation of ubiquitinated proteins. Ubiquitin proteasome system activity is integral to estradiol’s effects on memory, then this could lead to exciting new avenues of basic research into hormonal regulation of cognition, which could lead to important clinical implications for treating neurodegenerative disorders in which sex-based differences play a role [55]. From above analysis, it can be concluded that 13 genes were potential biomarkers for pathogenesis of AD. Assessing and confirming the clinical utility, we constructed a 15-marker panel (13 genes combined with age and sex) based SVM model, which can effectively predicts AD in independent testing sets.

Our work differs most significantly from previous work in the following ways: 1) Here, for the first time, we show the molecular mechanisms of sex differences in peripheral blood from Alzheimer's patients. 2) To identify the diagnostic value of these genes, we used machine learning methods to reduce overfitting and it can be improved in future studies.

Our study has some limitations: 1) Although we registered a second dataset for external verification, our results need to be confirmed by larger-scale studies. 2) The group differences in education and body mass index are potential confounds that could have affected our results, so further research is needed to examine the risks for these subgroups. 3) The mechanisms of these hub genes remain undefined in AD and they could be further explored to elucidate the functions and underlying mechanisms.

## Conclusion

Our analyses revealed different hub genes of AD by the analysis of DEGs for males and females separately. We find that the pathophysiological pathways of AD differ in males and females. Using 13 genes as a base, we developed a diagnostic model with a high AUC value in peripheral blood This study provides insight into the underlying molecular mechanisms for sex dimorphism as well as potential biomarkers that may be useful for diagnostics and therapy.

## Supplementary Information

**Additional file 1:** **Supplementary Data 1.** List of DEGs in females.

**Additional file 2:** **Supplementary Data 2.** List of DEGs in males.

**Additional file 3:** **Supplementary Data 3. **Intersected DEGs lists of males and females.

**Additional file 4:** **Supplementary Data 4. **Significantly enriched GO entries and KEGG pathways in females.

**Additional file 5:** **Supplementary Data 5. **Significantly enriched GO entries and KEGG pathways in males.

**Additional file 6:** **Supplementary Data 6. **GSEA in females.

**Additional file 7:** **Supplementary Data 7. **GSEA in males.

**Additional file 8:** **Supplementary Data 8. **Degree, MNC, Radiality, Stress and Closeness were used to screen 11 overlapping hub genes in females.

**Additional file 9:** **Supplementary Data 9. **Degree, MNC, Radiality, Stress and Closeness were used to screen 8 overlapping hub genes in males.

**Additional file 10:** **Supplementary Data 10.** A statistical analysis of immune checkpoint genes and 13 hub genes.

## Data Availability

The datasets generated and/or analyzed during the current study are available in the [GSE63060] repository [https://www.ncbi.nlm.nih.gov/geo/query/acc.cgi?acc=GSE63060], in [GSE63061] repository [https://www.ncbi.nlm.nih.gov/geo/query/acc.cgi?acc=GSE63061].

## Abbreviations

AD: Alzheimer's disease
GEO: Gene Expression Omnibus
DEGs: Differentially expressed genes
SVM: Support Vector Machine
GO: Gene Ontology
KEGG: Kyoto Encyclopedia of Genes and Genomes
MCI: Mild cognitive impairment
CSF: Cerebrospinal fluid
BP: Biological Process
CC: Cellular Component
MF: Molecular Function
PPI: Protein–protein interaction
MNC: Maximum Neighborhood Component
GSEA: Gene set enrichment analysis
AUC: Area under curve
